# Testing automatic methods to predict free binding energy of host–guest complexes in SAMPL7 challenge

**DOI:** 10.1007/s10822-020-00370-6

**Published:** 2021-01-19

**Authors:** Dylan Serillon, Carles Bo, Xavier Barril

**Affiliations:** 1 Laboratoire de Synthèse des Assemblages Moléculaires Multifonctionnels, Institut de Chimie de Strasbourg, CNRS/UMR 7177, Université de Strasbourg, Strasbourg, France; 2 Institut de Biomedicina de la Universitat de Barcelona (IBUB) and Facultat de Farmacia, Universitat de Barcelona, Av. Joan XXIII 27-31, 08028 Barcelona, Spain; 3 Institut Català d’Investigació Química (ICIQ), The Barcelona Institute of Science and Technology, Av. Països Catalans, 17, 43007 Tarragona, Spain; 4 Catalan Institution for Research and Advanced Studies (ICREA), Passeig Lluis Companys 23, 08010 Barcelona, Spain

**Keywords:** Molecular dynamics, Molecular mechanics, Semi-empirical methods, Machine learning, Computational drug design, Binding free energy calculations, Xtb GFN2B

## Abstract

The design of new host–guest complexes represents a fundamental challenge in supramolecular chemistry. At the same time, it opens new opportunities in material sciences or biotechnological applications. A computational tool capable of automatically predicting the binding free energy of any host–guest complex would be a great aid in the design of new host systems, or to identify new guest molecules for a given host. We aim to build such a platform and have used the SAMPL7 challenge to test several methods and design a specific computational pipeline. Predictions will be based on machine learning (when previous knowledge is available) or a physics-based method (otherwise). The formerly delivered predictions with an RMSE of 1.67 kcal/mol but will require further work to identify when a specific system is outside of the scope of the model. The latter is combines the semiempirical GFN2B functional, with docking, molecular mechanics, and molecular dynamics. Correct predictions (RMSE of 1.45 kcal/mol) are contingent on the identification of the correct binding mode, which can be very challenging for host–guest systems with a large number of degrees of freedom. Participation in the blind SAMPL7 challenge provided fundamental direction to the project. More advanced versions of the pipeline will be tested against future SAMPL challenges.

## Introduction

Supramolecular chemistry has experienced enormous growth in recent years. Supramolecular processes, and host–guest systems in particular, are studied both from a fundamental perspective and for their possible applications [1–4]. By improving the stability or modifying the properties of an encapsulated compound, or even by enhancing binding selectivity, we foresee a wide range of opportunities that span from industrial processes [5] to the medical field [6], such as drug delivery targeting cancer cells [7]. At the moment, breakthrough discoveries in supramolecular chemistry are hampered by the complexity of the thermodynamic and kinetic characterization of the inclusion/release processes, which make it difficult to generate useful predictions about molecular encapsulation [8]. Quantitative predictions of binding free energies are particularly difficult, but essential to guide the synthetic efforts, leading to more efficient design and discovery of host–guest systems with the desired activities. In this context, some of the tools currently used in computer-aided drug design (CADD) could be as useful for this endeavor, in the same way as they are for drug discovery [9–12]. At the same time, host–guest systems are orders of magnitude simpler (in terms of degrees of freedom) than biomolecular complexes and, because they are chemically stable, can be studied in a variety of well-controlled environmental conditions. This offers an opportunity to test and validate computational methods before they become part of the CADD arsenal.

Several years of SAMPL (Statistical Assessment of the Modeling of Proteins and Ligands) challenge have shown interesting approaches to compute the binding free energy of host–guest complexes, with a relatively large range of methods and performances [3–8]. In the context of the NOAH European Network, our interest is to develop automated methods for the prediction of arbitrary host–guest systems under different solvation and other environmental conditions. To fulfill this task, our focus is on automation (to enable users without a computational chemistry background), throughput (to deliver fast predictions and enable multiple concurrent users), and accuracy (to deliver useful predictions). We are not tied to any particular method, but must be able to generate predictions for a wide range of systems, including both novel and previously-described host systems.

In any computational strategy for structure-based design, an important step is the prediction of probable conformations of the receptor-ligand complex [13–15]. As a compromise between performance and accuracy, here we explore molecular docking (optionally refined with molecular mechanics) and molecular dynamics to solve the docking problem. Then, the complex can be scored with a variety of methods [16–19]. We explore the use of machine learning, which can be accurate and very efficient, but is limited by the amount of pre-existing data. As a complementary tool that can be applied to any arbitrary host–guest system, we also implement a physics-based method. In particular, recent progress in semi-empirical methods lead us to build MMGBSA-like methodology [19], but using the xtb software and GFN2B basis set [20] instead of a molecular mechanics forcefield.

At this stage, more important than the actual predictions, participation in the SAMPL7 was extremely useful to test several ideas in blind mode, resulting in the design of an automatic pipeline that will be evaluated in subsequent SAMPL challenges and eventually become publicly available.

## Methods

### Docking

Docking is used to generating a first guess of the Host–Guest structure. We chose ADV for our assessment for several reasons. It (i) is faster and generally performs better than AutoDock itself, (ii) is freely available and competitive with commercial tools [21, 22].

Docking was performed using AutoDockVina v. 1.1.2 [23]. Input comprises the host system, guest, and docking box, while the output is a list of poses ranked by ΔG_bind_, the predicted binding energy in kcal/mol (‘score’ =  − ΔG_bind_). To obtain the maximum number of poses, we set *num_modes* to 20. Three different box sizes are used, all of them cubic and centered in the host cavity: one big box (edge length 10 Å) that allows completely blind docking, and two small boxes (7 Å and 5 Å, respectively) restricted to the expected binding site. For each box, the top-scored solution is extracted. Additionally, for the smallest box, the extraction is followed by a steepest descent [24] and conjugated gradient [25] minimization to correct any ligand distortion caused by the small size box.

### Molecular modeling/molecular dynamic

All molecular dynamic (MD) simulations were set up in two steps:

1. *Host and guest preparation* The antechamber and tleap programs from the AMBER 18 package [26] are used to parameterize and solvate the system, respectively. Charges are derived with the AM1-BCC method [27]. For the spontaneous association simulations (SaMD), the host and the guest are placed in the same box, but not in direct contact, to observe their interaction preferences over time. Each system was solvated with ~ 2000 TIP3P water [28] in a cubic box whose dimensions were defined by a distance of 12 Å between the complex and the edges.
2. *Minimization* The system is minimized with GROMACS [29–34] using the steepest descent algorithm, then equilibrated for 100 ps with the leapfrog integrator.

Production simulations were run also with GROMACS in the NPT ensemble with temperature control using V-rescale thermostat [35] at 300 K and with tau_t = 0.1 and pressure control provided by Berendsen barostat [36]. Note that the Nosé-Hoover or Parrinello-Rahman barostats [37] are considered a better option for simulations at equilibrium, but as the MD is used to sample conformations (rather than extracting thermodynamic properties) this choice does not hamper the quality of the results. The Verlet-cutoff-scheme [38] is used and the frequency to update the neighbor list is initially set at 10, whereas long-range electrostatics were handled with the PME [39] method with PME order set at 4.0. 500 ns of simulation are realized for the systems, saving a snapshot every 0.1 ns for a total of 5000 frames for each simulation.

### Semi-empirical calculations

We used the xtb program package (version 6.1) [20] to calculate both the energy and the enthalpic and entropic corrections. It uses the GFN2B parametrization on an extended semiempirical tight-binding model, which has shown to be efficient for determining structures and noncovalent interaction energies for large molecular systems (in the order of 1000 atoms) [40–43]. Water solvent effects were included through a Generalized Born (GBSA) model. The convergence criteria thresholds were set as *extreme*. Optimization, followed by hessian calculations were performed. The resulting geometries were verified as true minima by checking that no imaginary vibrational frequencies remained. The temperature was set to 298.15 K for assessing the thermostatistical corrections.

### Machine learning

For the GDCC-7 dataset in SAMPL7, we decided to test a machine learning approach, taking advantage of pre-existing data. All OA or TEMOA host–guest systems from previous SAMPL challenges (SAMPL4 to SAMPL6) were collected, reconstructed from 2D to 3D, and optimized with the GFN2B method. Then, we used the CORINA web-platform [44] to compute 200 2D and 3D molecular descriptors for each system.

The descriptors of the dataset are reduced using the R software [45] with different approaches: (a) deleting the descriptors that have a near-zero variance; (b) deleting the most correlated descriptors using Caret package [46]; (c) using principal component analysis (PCA) [47] to combine descriptors that explain the most the variability.

In order to predict the binding free energy, several machine learning models using regression are used: neural network [48], knn [48], polynomial SVM [49], and random forest [50]. By modifying the parameters on those ML models, hundreds of different models are generated. In all cases we use a data partition of 30/70, resulting in a set of 26 cases for training and 8 cases for the test set. Our best model used to make predictions on SAMPL7 is a neural network using the "nnet" function, which provided an RMSE of 0.92 kcal/mol and with MAE about 0.85 kcal/mol, suggesting that the prediction is not excessively biased by overtraining.

## Results and discussion

### Thermodynamic-based approach

The Gibbs free energies of the optimized geometries were calculated as the sum of the Electronic Energy (E), which includes the D4 dispersion correction, thermostatistical corrections (G_RRHOT_) calculated following a coupled rigid-rotor-harmonic-oscillator approach, and the solvation contribution (G_solv_) calculated by the implicit solvation model GBSA.

$$\Delta G=E+{G}_{RRHOT}+{G}_{solv}$$

with

$${\Delta G}_{solv}={\Delta G}_{born}+{\Delta G}_{sasa}+{\Delta G}_{hb}+{\Delta G}_{shift}$$

The association Gibbs free energy is calculated from the difference of the free energies from the complex, host, and guest molecules, each on their respective conformational minimum.

$${\Delta G}_{bind}={\Delta G}_{complex}-{\Delta G}_{host}-{\Delta G}_{guest}$$

**Fig. 1:**
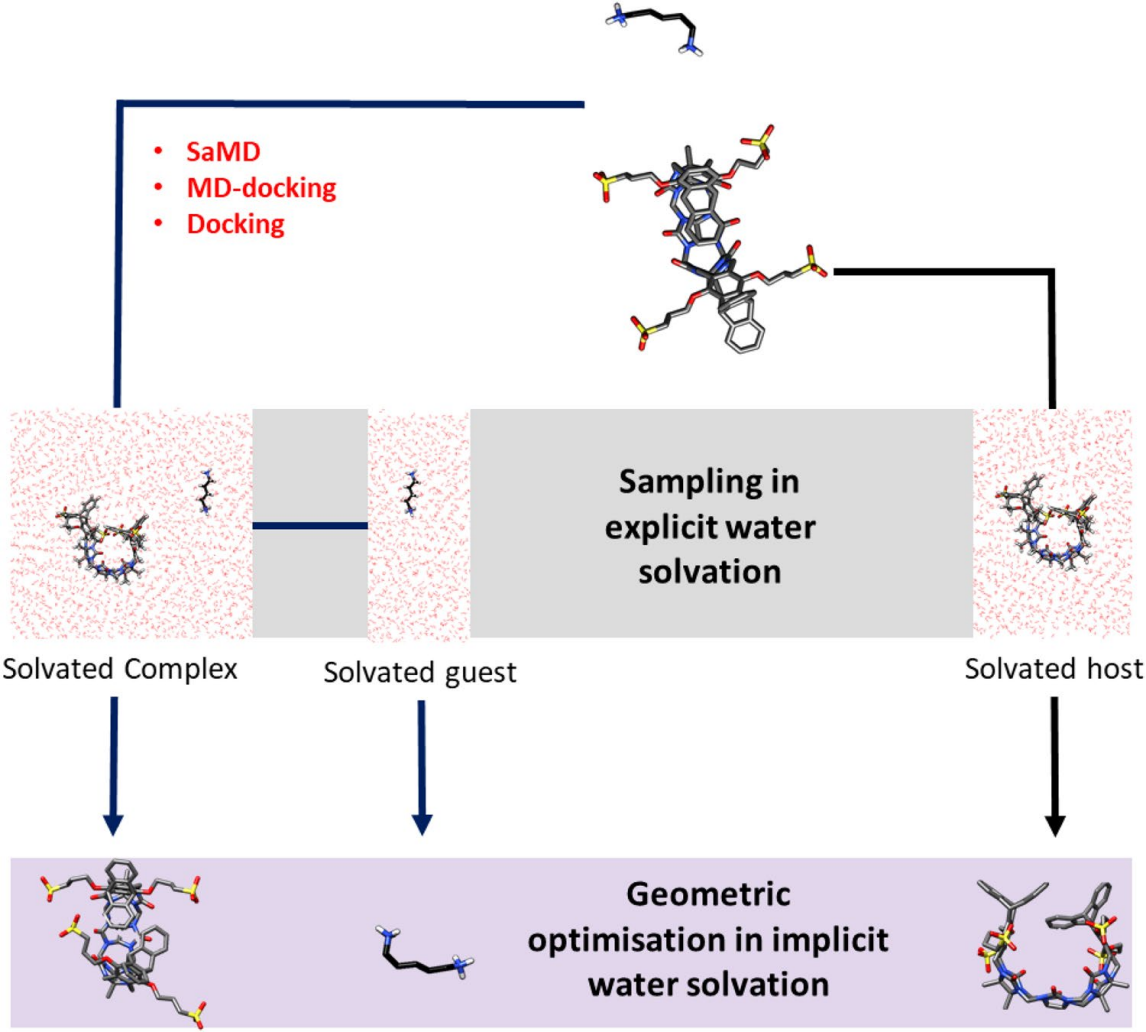
Protocol used to generate low-energy conformations of the apo host, the guest, and the host–guest systems. Three methods have been tested to generate initial models of the host–guest complex: SaMD, MD-Docking, and Docking. MD with explicit aqueous solvation is used to sample the conformational space. Then, for representative conformations, water is deleted and the geometry is minimized with the GFN2B basis set in GBSA implicit water solvation

Considering the complexity of the conformational energy landscape of the complex and host molecule, we used multiple geometries of the unbound host system as starting points for minimization, thus increasing the probability of finding the absolute minimum. To do so, we extract approximately 15 structures from the classical molecular dynamics simulations and carry out a geometric optimization at a semi-empirical level, followed up by calculation of the hessian to confirm that the final energy is a true minimum (i.e. all vibrational frequencies are positive). The variation in free energy was as large as 10 kcal/mol for the different geometries, which confirmed the importance of conformational sampling. The overall lowest energy structure was defined as a reference for free energy calculation. Though the degrees of freedom of the guests are much reduced, we use a similar protocol for consistency (Fig. 1).

### Retrospective analysis of trimertrip

As a proof of concept for our methodology, we used the data from the trimertrip set in the SAMPL3 challenge. This host is similar but simpler than the one in SAMPL7. Docking with a large box (15 Å^3^) produced complexes with negative binding energy (scoring), but the guest only formed surface interactions with the host. This led us to test two additional docking conditions where the docking space is progressively reduced. The resulting docking geometries have positive scores, indicative of conformational clashes, but in this case, the guest inserts into the host cavity. Three to five different binding modes were selected for each docking protocol. Minimization with Chimera (see “Methods”) allowed the system to relax before minimization and free energy calculation with xtb-GFN2B. Interestingly, the lowest-energy binding mode originated from the most restrictive docking protocol.

**Fig. 2:**
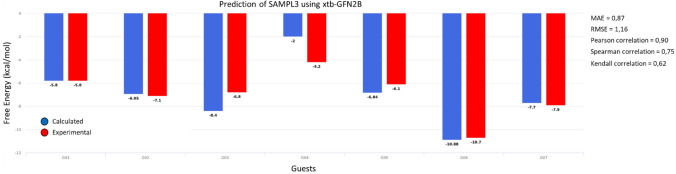
Results on the retrospective analysis of SAMPL3 Host–Guest complexes. Free energy predictions (blue bars) and experimental values (red bars) are in excellent agreement

As shown in Fig. 2, the predicted binding free energies are in excellent agreement with the experiment [RMSE = 1.16 kcal/mol; MAE = 0.87 kcal/mol; Pearson’s correlation [51] (r) = 0.90; Spearman’s rank [52] correlation (ρ) = 0.75, Kendall’s tau correlation [53] = 0.62(τ)]. In fact, in four out of the seven test cases we obtain quantitative agreement. In one case the error is below 1 kcal/mol and in the two remaining cases, the errors are 1.6 kcal/mol and 2.2 kcal/mol. This led us to believe that, given the correct binding mode, the GFN2B semiempirical method could provide QM-level results at a small fraction of the computational cost (minimization plus calculation of the vibrational frequencies takes 1 to 2 h per geometry on a desktop computer).

For that specific SAMPL3 dataset, retrospective analysis of the results shows very accurate results compared to the ones that have been published initially [54].

### SAMPL7 trimer-trip binding mode generation

**Fig. 3:**
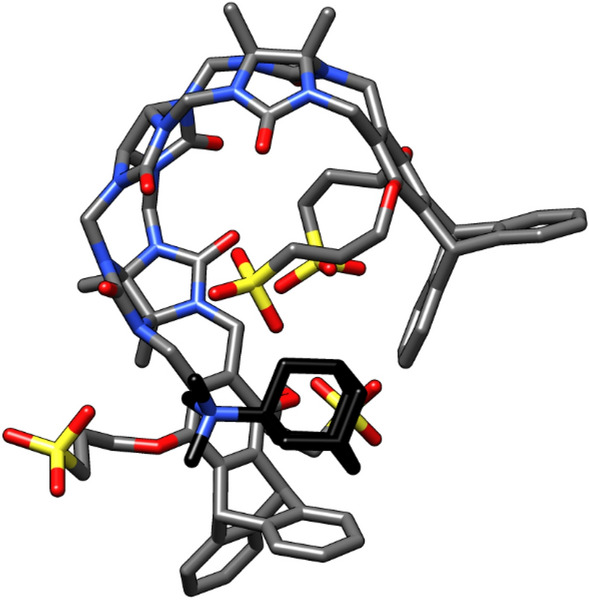
Binding mode of guest molecule G06 generated with docking and xtb. A sulfonate group enters the host pocket during geometric optimization, revealing an inadequate balance of solvation terms

As in the test systems above, host–guest interactions were predicted by molecular docking considering different docking volumes in order to obtain a variety of binding modes, including somewhere the guest is fully inserted into the host. In the most restrained volume (which forces the guest to be located inside the host but yields positive score values), a molecular mechanics (MM) minimization of the docking solution is performed with MOE and CHIMERA, thus removing any potential clash between host and guest. For some particular systems (G08 and G10) the MM minimization was deemed insufficient to attain a relaxed complex. In those cases, docking was followed by 200 ns of MD simulations. Even then, it failed to generate any binding mode where the guest is embedded into the cavity of the cyclic host. Further adding to our problems, the sulfonate groups tended to form unrealistic interactions after minimization with xtb. In some cases, the sulfonates were even inserted into the host pocket, which is largely hydrophobic, instead of remaining solvent-exposed, as expected for a negatively charged group (Fig. 3). This indicated that the implicit solvation model in xtb underestimates the desolvation cost of ionic groups.

Contrary to what was observed with the trimer-trip host–guest systems of previous editions, we had to conclude that a better method was necessary to generate correct binding modes for the SAMPL7 test set. Our method should allow for host flexibility in order to allow guest embedding with reasonable geometries. On the other hand, it was clear that the implicit solvation model implemented in xtb was falling short for ionic systems, and explicit solvation would be necessary for the conformational sampling stage. Both requisites pointed to MD simulations as an optimal solution, which we proceeded to implement and test.

**Fig. 4:**
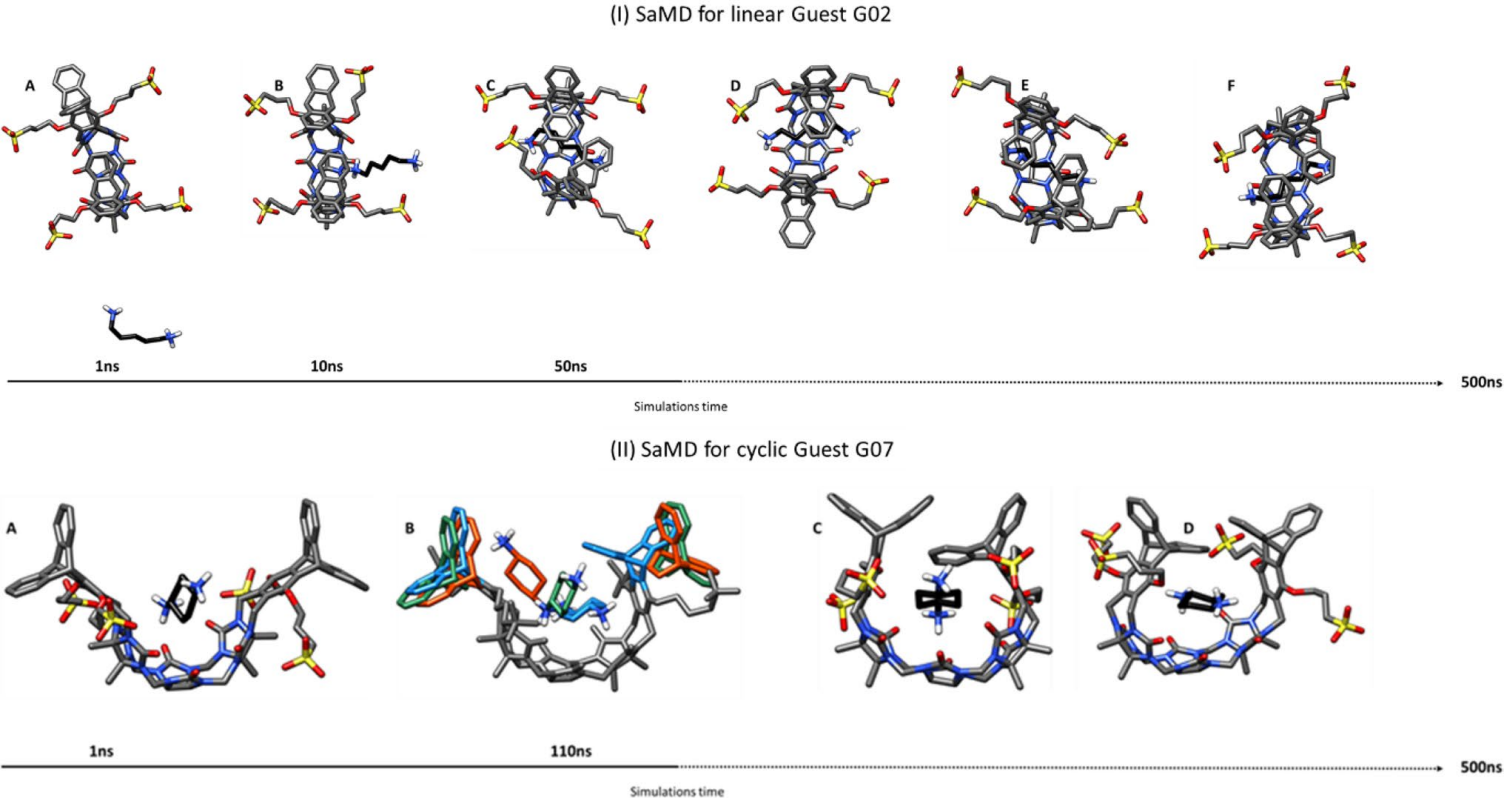
Inclusion process for trimer-trip dost-guest complexes observed with SaMD. (I) Linear guest G02 **a** starts from a fully dissociated state; **b** after ~ 10 ns, surface interactions are formed between host and guest; **c** eventually, the host widens the cavity and the guest molecule slides across to form a complex; **d**–**f** the complex remains stable but explores a variety of conformations for the remaining of the simulation. (II) Cyclic guest G07 **a** forms an encounter complex very early (~ 1 ns); **b** and remains in contact with the host for over 100 ns, until the host clicks into the closed geometry; **c**, **d** the complex remains stable but explores a variety of conformations for the remaining of the simulation

In what we refer as Spontaneous association MD (SaMD), the host and the guest are simulated in a TIP3P water box, starting from a dissociated configuration (distance ≥ 8 Å), extending the simulation until binding is observed. For the linear guests G01, G02, and G05, SaMD successfully completed the inclusion process, which proceeded in two steps: (i) rapid formation of surface contact between host and guest, leading to stable interactions; and (ii) a small opening of the host system, enabling the entry of the guest into the host cavity and formation of a stable complex (Fig. 4). The second step is the bottleneck in the process. It occurs in a simulation time of 50 ns to 500 ns for the G01 compound, but for systems with longer alkyl chains (more degrees of freedom) takes a much longer time. In G05, for instance, the simulation had to be extended to 1 µs to observe a single association event (ca. 700 ns). The application of the same methodology to the cyclic guest (i.e. G06, G07, G08, G09, G10, G11, G18, G19) failed to produce correct binding modes. While the compounds form stable surface interactions, they do not enter the host. This is in line with the above observation that the host opening to admit the guest is the bottleneck in the association process. The bulkier nature of the cyclic guests implies that the host must (transitorily) adopt a wide-open conformation that is energetically unfavorable and cannot be sampled in the relatively short timescale of the MD simulations. To confirm this hypothesis, for the cyclic guest G07 we carried out an MD simulation starting from a fully open host system (generated by geometrical optimization in vacuum). The guest rapidly proceeds to interact with the (now exposed) interior of the host, forming a stable but dynamic binding mode. After approximately 100 ns, the host folds, trapping the guest in its interior (Fig. 4).

This result indicates that starting from metastable host conformations may be a general strategy to accelerate SaMD and generate valid host–guest geometries.

Notably, the binding mode of the guests inside the host is very dynamic, with fast rotations and frequent sliding movements that are only limited by the resistance of the charged group of the guest to enter the hydrophobic core of the host. As expected, the ionic groups rarely form direct contacts. Instead, they preserve their solvation shells. Overall, these results suggest that SaMD is an optimal and feasible strategy not only to obtain a bound conformation of the host–guest complex but also to capture the rich conformational diversity of the bound state. Unfortunately, between the setting up and testing of this protocol and the computational cost of the MD simulations, it was impossible to complete all these calculations by the challenge deadline. Posterior analysis confirms that correct identification of the binding mode through SaMD improves the quality of the binding free energy predictions (see next section).

### SAMPL7 trimer-trip free energy prediction

For each complex, we extract 5 to 10 different binding modes generated with the above-described protocols. These geometries are then individually minimized at the xtb-GFN2B semi-empirical level, and only those yielding a true minimum (i.e. all vibrational frequencies are positive) are considered. The lowest energy complex is considered as the true minimum, except for a few cases where visual inspection identified issues with the corresponding geometry, always related to inadequate screening of charges by the implicit solvation method, such as those shown in Fig. 3. Predictions for each system are shown in Table 1.

**Table 1:** Final results with experimental, calculated free binding energy and the error related

Challenge system	Case name	Experimental ΔG_bind_ (kcal/mol)	Predicted ΔG_bind_ (kcal/mol)	Error (kcal/mol)
TRIMERTRIP	G01	− 6.10	− 5.50	− 0.60
	G02	− 8.32	− 8.30	− 0.02
	G03	− 10.05	− 5.40	− 4.65
	G05	− 11.10	− 8.30	− 2.80
	G06	− 9.60	− 2.50	− 7.10
	G07	− 6.50	− 6.10	− 0.40
	G08	− 9.45	− 11.50	2.05
	G09	− 7.57	− 2.00	− 5.57
	G10	− 8.17	− 8.40	0.23
	G11	− 9.02	− 1.30	− 7.72
	G12	− 8.29	− 2.90	− 5.39
	G15	− 10.52	− 6.70	− 3.82
	G16	− 11.50	− 7.10	− 4.40
	G17	− 11.80	− 6.40	− 5.40
	G18	− 10.55	0.00	− 10.55
	G19	− 11.70	0.00	− 11.70
GDCC	OA-G1	− 4.97	− 4.95	− 0.02
	OA-G2	− 6.91	− 7.79	0.88
	OA-G3	− 8.10	− 8.26	0.16
	OA-G4	− 6.76	− 7.33	0.57
	OA-G5	− 4.73	− 4.50	− 0.23
	OA-G6	− 4.97	− 4.92	− 0.05
	OA-G7	− 6.07	− 5.81	− 0.26
	OA-G8	− 8.25	− 6.12	− 2.13
	ExoOA-G1	0.00	− 5.67	5.67
	ExoOA-G2	− 2.20	− 4.75	2.55
	ExoOA-G3	− 3.37	− 6.60	3.23
	ExoOA-G4	− 3.61	− 7.10	3.49
	ExoOA-G5	− 5.57	− 3.91	− 1.66
	ExoOA-G6	− 5.83	− 5.92	0.09
	ExoOA-G7	− 6.98	− 5.67	− 1.31
	ExoOA-G8	− 7.67	− 6.14	− 1.53

For guest G18 and G19 we could not find a correct binding mode SaMD, and the docking results gave positive binding energy. As both protocols failed for these two cyclic guests (presumably due to their large volumes) we desisted from making predictions for them.

**Fig. 5:**
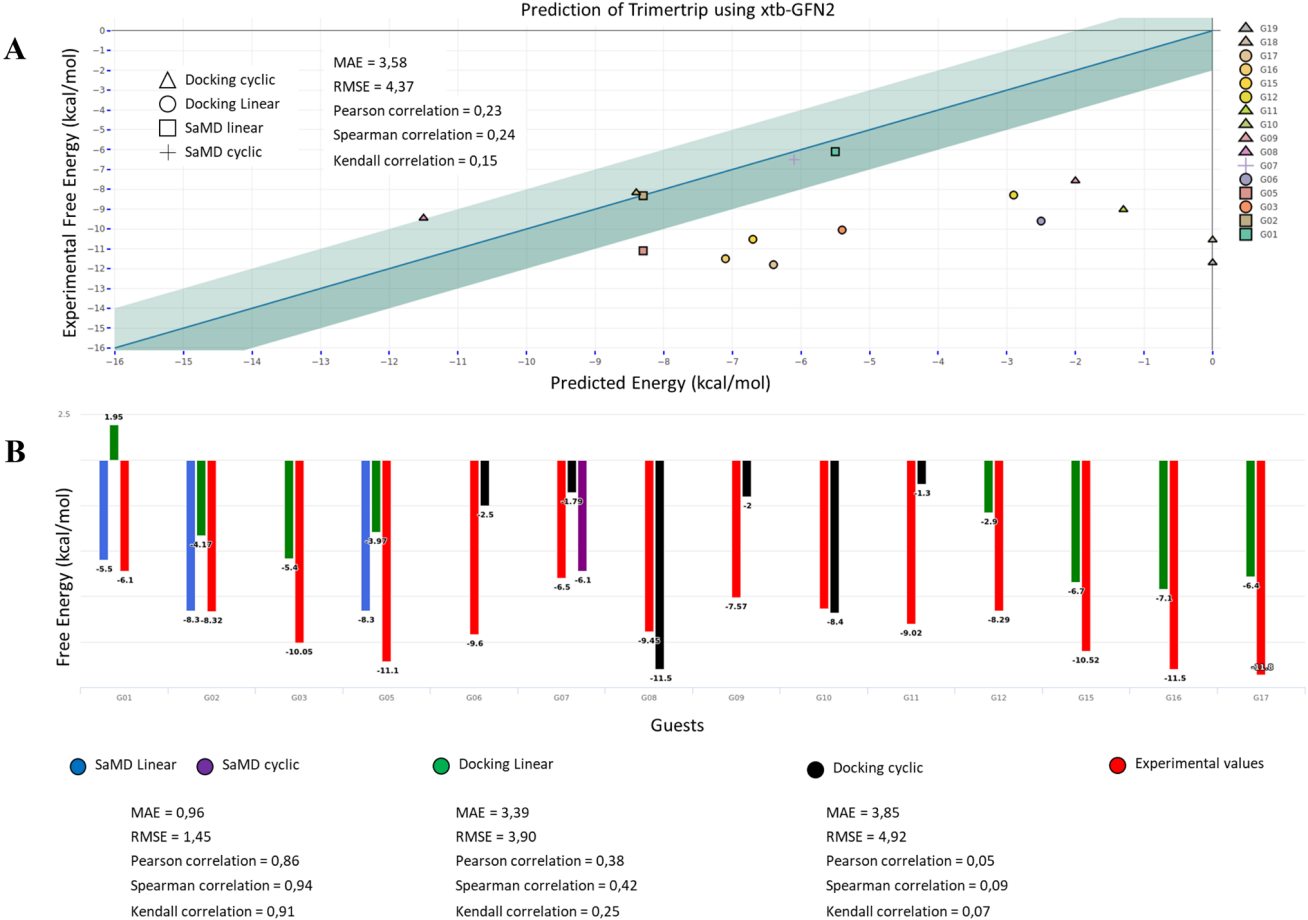
Comparison of experimental binding free energies with predicted values. (Top) correlation plot; the green-shaded area represents a threshold of + 1/− 1 kcal/mol from the experimental energy; the symbols indicate the nature of the guest and the method used for binding mode generation (triangle = docking for cyclic guest, circle = docking for the linear guest, square = SaMD for the linear guest, cross = SaMD for cyclic guest). (Bottom) histogram of free binding energy colored by the method used for binding mode generation (black = docking for the cyclic guest, green = docking for the linear guest, blue = SaMD for Linear guest, purple = SaMD for cyclic guest). G18 and G19 guests are not shown or consider for statistical analysis because it was not possible to generate a plausible binding mode for them

We can see in Fig. 5a, three different zones in the graphics: The first zone corresponds to the five host–guest systems that have been predicted well. Concerning these systems G01, G02, and G07 are extracted from the SaMD protocol. While G08 and G10 are the two cyclic host from where interaction outside the cavity have been extracted from MD-docking. The second zone corresponds to five Host–Guest system where our prediction was incorrect, but still within a range from the experimental values (3 to 5 kcal/mol errors). These complex (G03, G05, G15, G16, G17), are mainly linear and the results originate from docking poses with the exception of G05, which originates from SaMD (result obtained after the submission deadline). The third zone corresponds to the six host–guest with large errors, including the G18 and G19 (for which none negative binding energy has been found). Most of them are cyclic and the errors can be attributed to our inability to find reasonable binding modes in the timeline of the challenge.

In Fig. 5b, we show that for the complexes where SaMD delivers a correct binding mode, the binding free energy predictions are far superior to the results obtained from docking poses. In fact, most cases (G01, G02, G05, G07) are in quantitative agreement with the experiment (± 1 kcal mol) and the overall performance statistics are excellent: for RMSE = 1.45 kcal/mol; MAE = 0.96 kcal/mol; Pearson’s correlation (r) = 0.86; Spearman’s rank correlation (ρ) = 0.94, Kendall’s rank correlation = 0.91(τ). Compared to SaMD, the results from docking underestimate the binding free energy, which suggests that lower-energy conformations of the Host–Guest complex can be sampled with MD, but not with the MM protocols.

### Knowledge-based approach

**Fig. 6:**
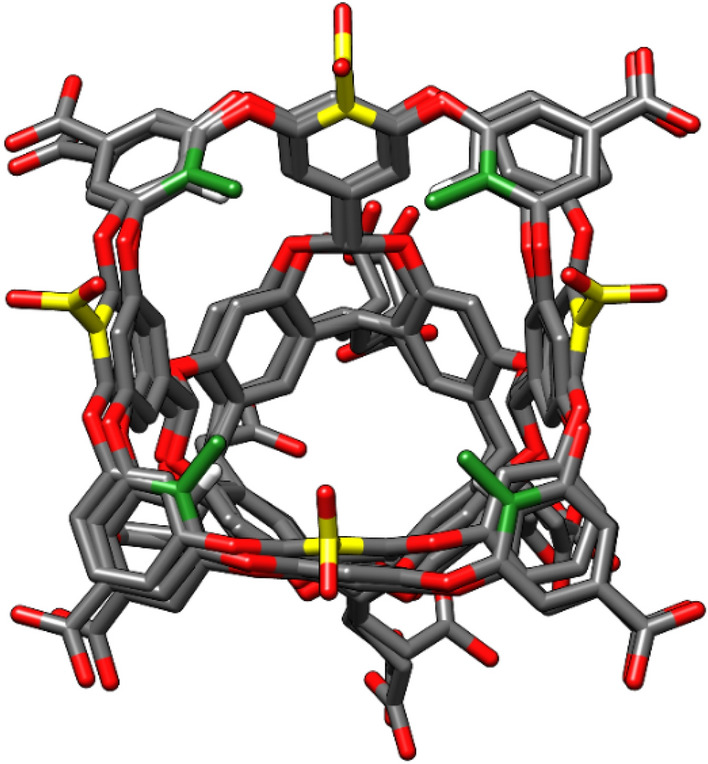
Three different systems are used for the GDCC prediction. In grey with have the common scaffold representing the major part of the host. The differences between the host are highlighted: the TEMOA system (SAMPL3-6) in green differing from OA (SAMPL3-6) by the methyl in green. The exoOA system (SAMPL7) differing from OA host by the addition of 4 carboxylate groups in yellow

**Fig. 7:**
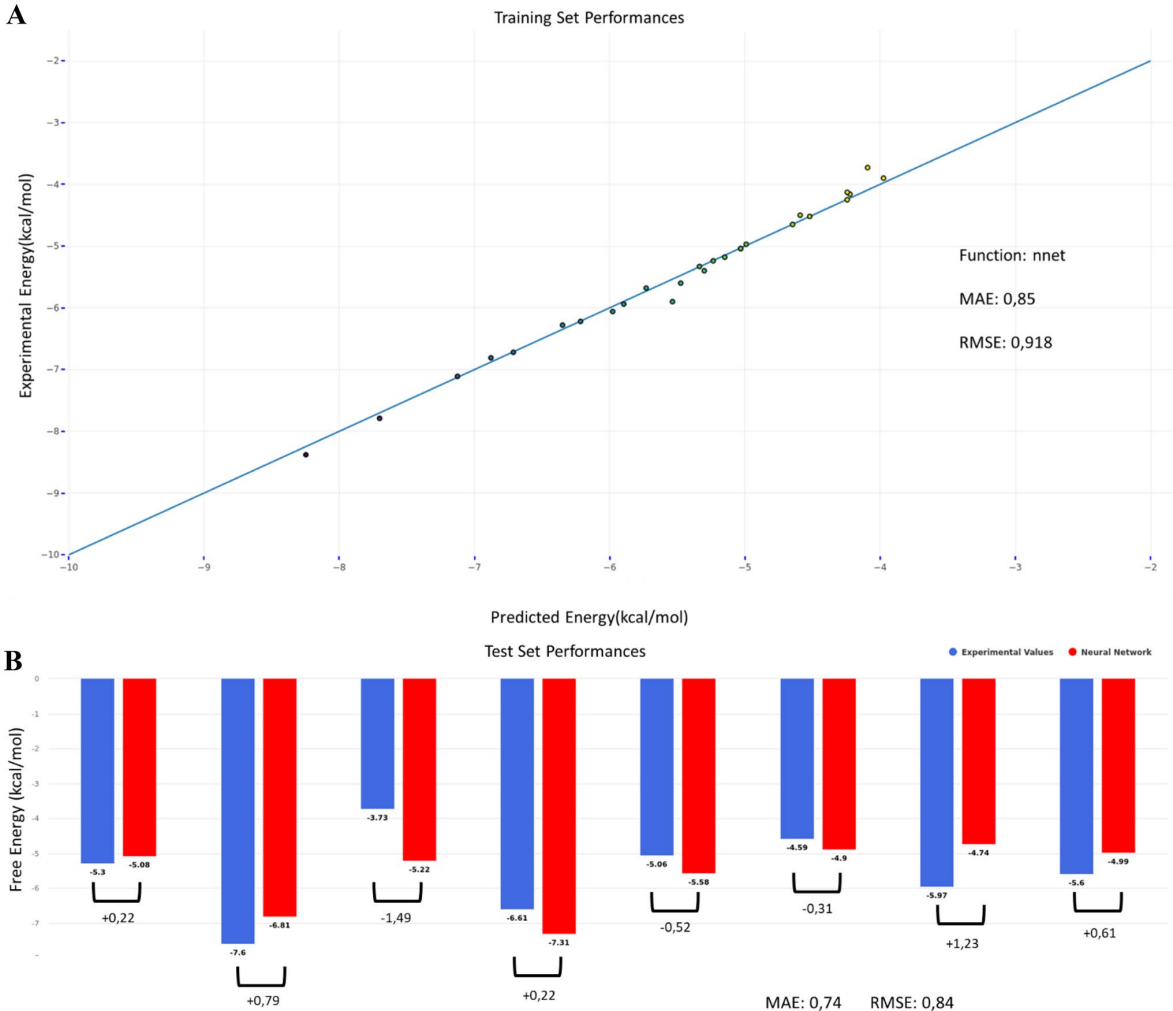
**a** Performance of the training set including 27 different guests interacting with two different systems. **b** The test set includes 8 guest molecules with free energy predicted using the training set

For GDCC prediction, as there was an important amount of pre-existing data from previous challenges, we decided to try an orthogonal approach-based ML. The dataset includes 35 compounds in total, belonging to three classes of host systems that are similar in structure and chemical composition: OA, TEMOA, and exoOA (Fig. 6). The binding free energy values range between − 3.73 and − 8.38 kcal/mol. The final model (see Methods) is a neural network, using 90 CORINA descriptors (60 describing the guest and 30 describing the host system). As expected, the predictions for the training set are very accurate, with RMSE = 0.92 kcal/mol and all the predicted values within a 1 kcal/mol range from the experimental values (Fig. 7a). For the test set, all the predicted values are close to the experimental one, with maximum and minimum errors of -1.49 kcal/mol and + 0.22 kcal/mol, respectively.

The GDCC-7 dataset to be predicted this year consisted of 8 guest compounds (4 charged and 4 non-charged) binding to two related host systems. After the model has been optimized, it takes only 10 s to calculate the free binding energy of the 8 guests in the 2 hosts. With RMSE and MAE values of 1.67 kcal/mol and 1.21 kcal/mol, respectively, the overall performance is rather satisfactory, especially by comparison with the thermodynamic-based approach. It is worth noting that the four negative guests are not predicting well, which can be explained by the limits of the model imposed by the composition of the training set: since the least favorable binding free energy value is − 3.73 kcal/mol, the model can’t predict more positive values. Even then, the hierarchy between the guest values is respected (G4 < G3 < G2). There is no experimental value for G1, so it has not been considered for this analysis. If we apply the same analysis to every subgroup (based on the positive or negative charge and the host they are interacting with) we obtain an almost perfect hierarchical prediction. The only exception is the OA-G7 complex, which was predicted lower than OA-G6 due to the fact that OA-G7 has been underestimated (− 5.67 kcal/mol instead of − 6.98 kcal/mol) while OA-G6 have been predicted very close to his experimental values (− 5.92 for − 5.83 experimental values).

**Fig. 8:**
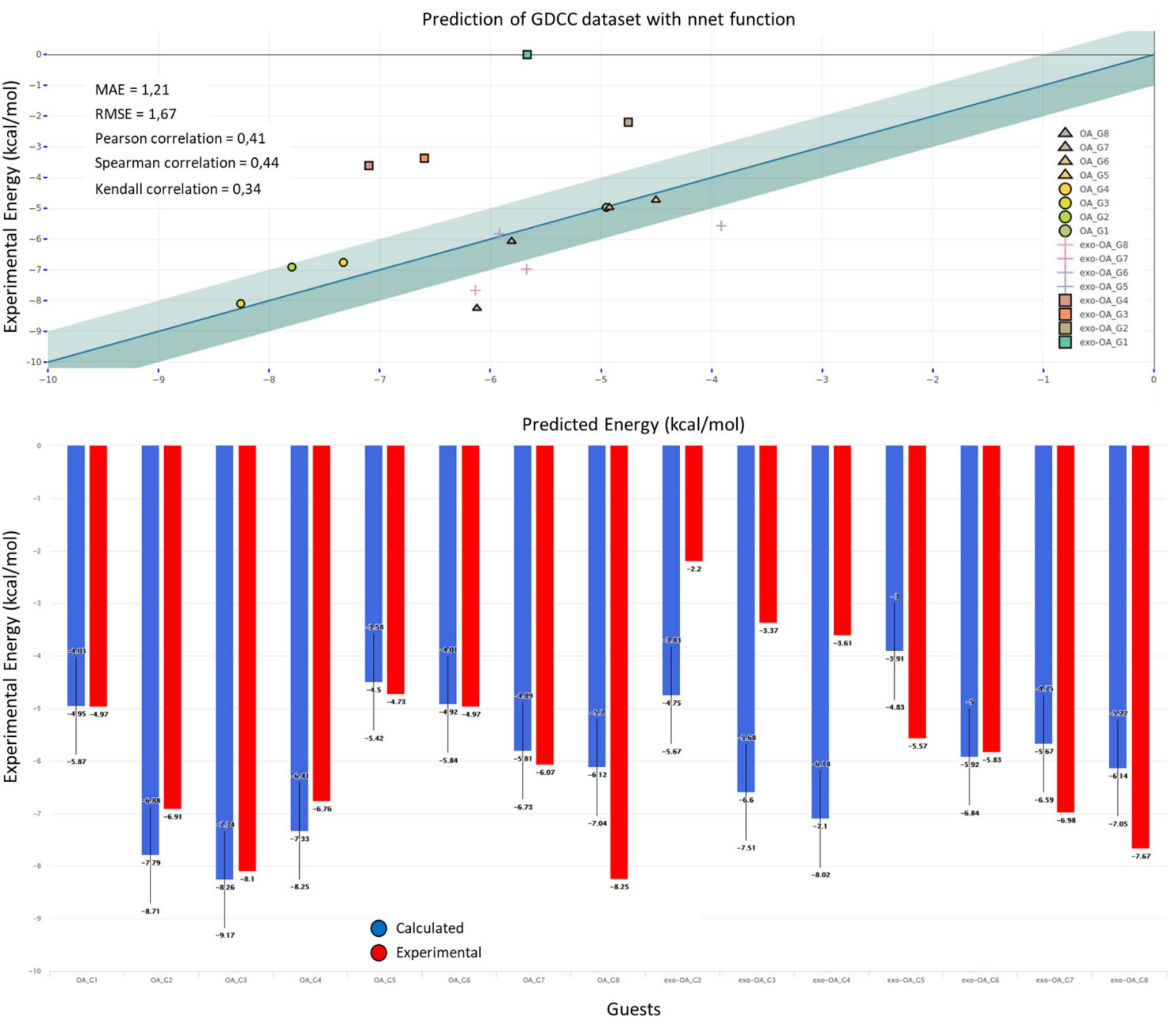
Comparison of experimental binding free energies with predicted values. (Top) correlation plot; The green-shaded area represents a threshold of ± 1 kcal/mol from the experimental energy; the symbols indicate the nature of the guest and each prediction has a different color (triangle = positively charged guest interacting with OA system, circle = negatively charged guest interacting with OA system, square = negatively charged guest interacting with the exo-OA system, cross = positively charged guest interacting with the exo-OA system). (Bottom) histogram of free binding energy with calculated (blue) and experimental values (red). The error bars reflect the RMSE of the nnet model on the training set (0.918 kcal/mol). As previously explained, there is no experimental value for G1, so it has not been considered for this analysis

In fact, all systems, except for the four negative compounds interacting with exo-OA, are predicted within 1 kcal/mol of the experimental values (Fig. 8). For the complexes involving the OA system, which features prominently in the training set, the predictions are better still, with MAE = 0.55 kcal/mol and RMSE = 0.85 kcal/mol.

## Conclusions

The participation in SAMPL7 allowed us to test two orthogonal approaches to calculate host–guest binding free energies, identifying in each case strengths and limitations that will be considered for the final design of an automated platform.

The thermodynamic-based approach is absolutely general and can be used, in principle, on any host–guest system. The use of an advanced semiempirical basis set (GFN2B) to calculate energies and thermostatistical corrections offers increased performance relative to MM approaches with a moderate computational cost (1–2 h on a single CPU) and eliminates the dependency on small-molecule force-fields, which are often inaccurate [55, 56]. However, we have identified two critical aspects that can lead to incorrect predictions. The first one is a critical dependency on the structure of the host–guest complex used to generate the prediction (the binding mode). For systems with significant host flexibility, rigid receptor docking can be inappropriate, and host conformational sampling is necessary. Direct observation of the host–guest pair formation through molecular dynamics with explicit solvent is an optimal solution in terms of quality of the binding free energy predictions but can be unpractical due to the long simulation times, which increase with the number of degrees of freedom of the system. Future implementations of the platform will consider modifications of the MD parameters to increase efficiency, including parallelization, GPU-based implementations, and/or various MD software. In the trimertrip case, we identified a slow transition between the closed and open conformation of the host as the bottleneck in the association process. For such cases, starting the SaMD simulations with open host conformations can yield excellent results at a fraction of the simulation cost. The second limitation of our approach is the implicit solvation method (GBSA) which can underestimate the desolvation cost of ionic species in aqueous solvation, leading to the formation of ionic pairs whose contribution is overvalued. Other reports have observed a systematic bias with implicit solvation models [57]. We do not observe such systematic bias, but the implicit solvation model remains one of the weaknesses of the approach. More recent xtb versions have replaced the GB formalism for an analytically linearized Poisson-Boltzmann (ALPB) model. It will be interesting to check the performance of ALPB in future SAMPL editions. In any case, the explicit solvation in MD simulations is better suited to preserve the solvation shells around the solute’s ionic groups. Thus, the use of MD snapshots as input geometries in xtb-GFN2B calculations seems to provide better results than exhaustive conformational sampling with implicit solvation.

The use of knowledge-based methods can be highly advantageous when there is sufficient pre-existing data. Contrary to protein–ligand complexes, where a large body of data exists, host–guest systems cannot benefit from massive training sets. Thus, we were particularly interested in examining the suitability of machine learning approaches, with a particular concern on the risk of overfitting. The results obtained on the GDCC system are really encouraging and motivate us to build a database of host–guest systems, with their corresponding binding free energies, and train both general and host-specific models. Two critical aspects that will be explored are the use of other molecular descriptors to improve the predictions and the introduction of selection criteria to decide when a particular system is within the scope of the model.

Overall, the participation in SAMPL7 has allowed us to design an automatic pipeline to compute binding free energies for any Host–Guest system. We currently implementing and improving the protocol, that will be tested in subsequent SAMPL editions.

### Results overview

See Table 1.

### Statistical analysis

See Table 2.

**Table 2:** Statistical analysis of SAMPL3 calculation, SAMPL7 TRIMERTRIP and SAMPL7 GDCC prediction

	MAE (kcal/mol)	RMSE (kcal/mol)	Pearson correlation	Spearman correlation (rho)	Kendall correlation (tau)
SAMPL3					
S3_completedataset (n = 7)	0.87	1.16	0.90	0.75	0.62
SAMPL7 TRIMERTRIP					
S7_complete dataset (n = 14)	3.58	4.37	0.23	0.24	0.15
S7_Linear (n = 8)	3.39	3.90	0.38	0.42	0.25
S7_cyclic (n = 6)	3.85	4.92	0.05	0.09	0.07
S7_confident (n = 4)	0.96	1.45	0.86	0.94	0.91
SAMPL7 GDCC					
S7_complete dataset (n = 15)	1.21	1.67	0.41	0.44	0.34
S7_OA (n = 8)	0.54	0.85	0.80	0.85	0.76
S7_exoOA (n = 7)	1.98	2.27	− 0.04	0.00	0.05
S7_positive_guest (n = 7)	1.56	2.08	0.74	0.89	0.81
S7_negative_guest (n = 8)	0.91	1.20	0.73	0.81	0.57
S7_Positive_OA (n = 4)	0.41	0.53	0.97	1.00	1.00
S7_negative_OA (n = 4)	0.67	1.08	0.90	1.00	1.00
S7_positive_exoOA (n = 3)	3.09	3.12	1.00	1.00	1.00
S7_negative_exoOA (n = 4)	1.15	1.31	0.68	0.80	0.67
